# Surgical repair of tetralogy of Fallot in a 78-year-old woman: a case report

**DOI:** 10.1186/s13256-024-04414-5

**Published:** 2024-03-04

**Authors:** Yuzo Katayama, Sho Isobe, Tsukasa Ozawa, Takeshiro Fujii

**Affiliations:** Division of Cardiovascular Surgery, Department of Surgery, Toho University Omori Medical Center, 6-11 Omori-Nishi Ota-Ku, Tokyo, 143-8541 Japan

**Keywords:** Tetralogy of Fallot, Adult congenital heart disease, Endocarditis

## Abstract

**Background:**

Tetralogy of Fallot is a congenital heart disease mostly diagnosed and treated in early childhood. However, there are some adult cases receiving treatment.

**Case presentation:**

We describe a 78-year-old Japanese woman who presented with severely hypertrophic right ventricle, ventricular septum defect, overriding aorta, and severe infundibular stenosis in the right ventricular outflow tract. As hypoxemia was mild and daily exertion was sufficiently possible, home oxygen therapy was introduced. After 1 month, she was referred because of a positive blood culture. The blood culture test was positive four times, therefore, the antibacterial drug was administered according to active infective endocarditis. SpO_2_ repeatedly decreased during hospitalization, thus oxygen was needed. As there were infective endocarditis onset and progressive hypoxemia, we planned a surgical correction.

**Conclusion:**

Tetralogy of Fallot was diagnosed and successfully treated with complete surgical correction, and the development of infective endocarditis was the definitive indication for surgery at this late age.

**Supplementary Information:**

The online version contains supplementary material available at 10.1186/s13256-024-04414-5.

## Background

Early surgical correction has been established as the best treatment for tetralogy of Fallot (TOF). Without surgical correction, most patients die during childhood, making adult patients with uncorrected TOF rare. Additionally, surgical indications for untreated adult TOF are mostly due to progression of heart failure [1]. We report an extremely rare case in which intracardiac repair was performed on an elderly cyanotic patient with infective endocarditis.

## Presentation of case

The case involved a 78-year-old Japanese woman diagnosed with ventricular septal defect (VSD) during her elementary school days. In her 40s, she felt exertional dyspnea fatigue, but was followed up without any restrictions. At 77 years, she consulted our adult congenital heart disease clinic for increasing symptoms since her 70s. She had a history of hypertension in her 40s and was taking oral calcium channel blockers (nifedipine; 10 mg) and angiotensin receptor blockers (ARBs; candesartan cilexetil; 4 mg) prescribed by a family doctor, and no other previous medical history. Although she had one pregnancy and birth, there were no special events when she gave birth. There was no history of smoking and drinking and no special cardiovascular events in her family history. She has been working as a typist in an office since her 20s.

**Fig. 1:**
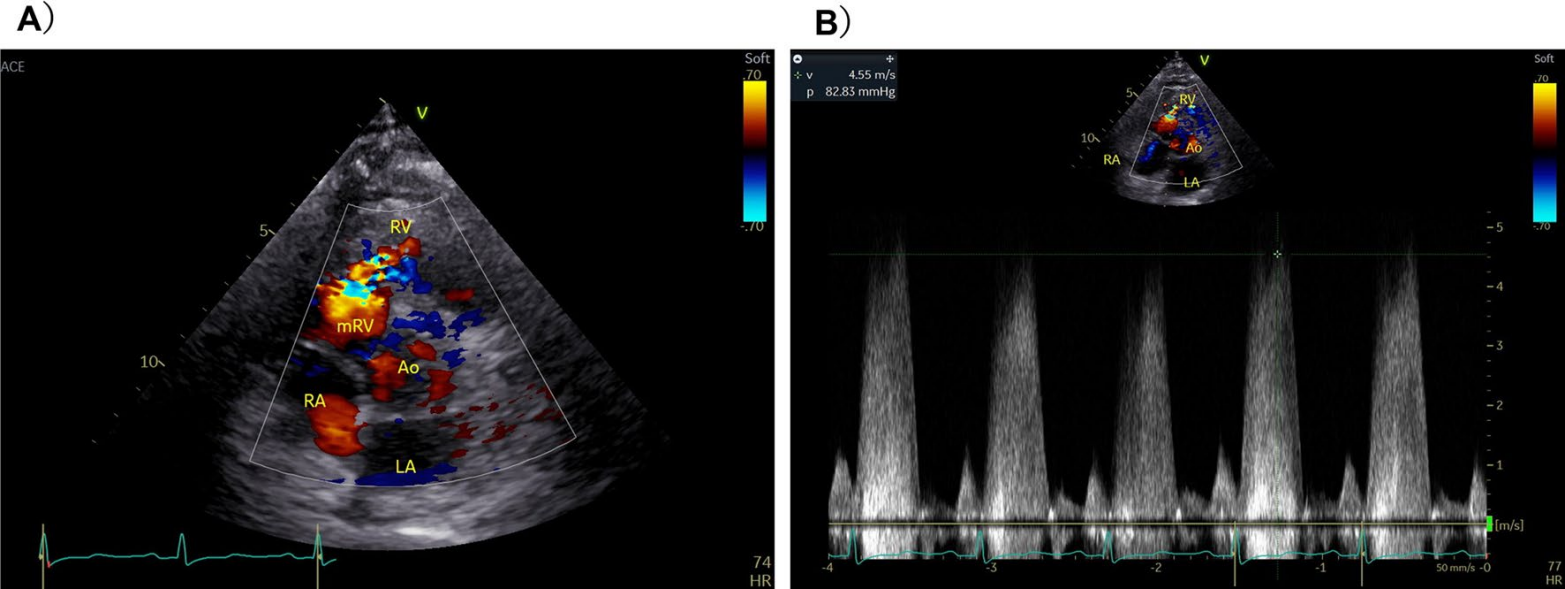
Transthoracic echocardiography revealing a ventricular septum defect in the perimembranous region and right ventricular outflow tract stenosis. **A** Color Doppler echo showing the short axis view. **B** Peak pressure gradient was 82 mmHg

Electrocardiography showed sinus rhythm, right bundle-branch block, and right ventricular hypertrophy with secondary ST–T wave abnormalities. Chest radiography showed an enlarged cardiac silhouette and dilated pulmonary arteries. Transthoracic echocardiography and contrast computed tomography (CT) revealed overriding aorta, right ventricular outflow tract (RVOT) stenosis (Fig. 1), and right ventricular hypertrophy in addition to VSD. The final diagnosis was tetralogy of Fallot (TOF). As hypoxemia was mild and daily exertion was sufficiently possible, home oxygen therapy was introduced after comprehensively explaining the disease and its prognosis.

After 1 month, she was referred from the family doctor and hospitalized because of a positive blood culture test. The physical findings on admission were as follows: body temperature 38.1 ℃, blood pressure 135/71 mmHg, pulse rate 71 beats per minute and regular, and SpO_2_ 87%. Systolic murmur was heard at the left sternal border of the second intercostal space, not rales. No special findings were found on the palpebral conjunctiva, fingers and toes, palms, and soles of the feet. No neurological abnormal findings were observed.

Laboratory findings on admission were as follows: WBC 11,000/μL, Hb 17.5 g/dL, Hct 54%, Plt 19.7/μL, CRP 4.1 mg/dL, AST 24 IU/L, ALT 13 IU/L, LDH 240 IU/L, Na 139 mEq/L, K 3.9 mEq/L, Cl 105 mEq/L, TP 7.0 g/dL, Alb 4.1 mg/dL, BUN 14 mg/dL, Cr 0.59 mg/dL, eGFR 73.1, BNP 88.4 pg/dL, urine specific gravity 1.015, PH 7.0, white blood cell count 0. The blood culture test was positive four times (Lactobacillus rhamnosus) and antibiotic susceptibilities of this bacterium were as follows; penicillin G > 2, aminobenzylpenicillin > 2, ceftriaxone 0.5, gentamicin 1, minocycline 0.25, vancomycin > 16, teicoplanin 16, and levofloxacin 2. Therefore, continuous intravenous administration of ceftriaxone 2 g/day was administered for 6 weeks according to active infective endocarditis. At the time of admission, the patient had a fever of 38 °C, but it remained at 36 °C 3 days after the start of administration. The inflammation data also normalized and negative blood cultures were confirmed twice 3 weeks after starting administration. SpO_2_ repeatedly decreased to 80% during hospitalization, thus oxygen was started and 3 L was needed to maintain around 90%. Transthoracic echocardiography, thoracic and abdominal CT, head magnetic resonance imaging (MRI), and upper and lower gastrointestinal endoscopy were also performed, but the site or route of infection could not be identified.

Cardiac MRI revealed severe infundibular stenosis and tiny pulmonary valve regurgitation (Additional file 1: Videos S1 and S2), normal right ventricular contractility, 90% normal left ventricular volume, and 0.67 Qp/Qs (pulmonary blood flow/systemic blood flow ratio). As there were progressive hypoxemia and infective endocarditis onset, we planned a surgical correction after infection control of infective endocarditis.

The operation was performed on full cardiopulmonary bypass. The hypertrophic muscle obstructing the RVOT was resected up to 20 mm and the VSD was closed with a patch. A perforation was found in the left semilunar cusp of pulmonary valve and the lesion accompanied by changes in curability was closed directly. The perforation may have been the source of infection. Intraoperative transesophageal echocardiography showed improvement in the RVOT to 2.5 m per second.

Although all follow-up blood culture tests after surgery were negative, ceftriaxone was administered for 4 weeks. The preoperative BNP level of 79 pg/dL increased to 727 pg/dL on day 8 postoperation, but improved to the same preoperative level after oral diuretic discontinuation, and echocardiography revealed normal cardiac function. The patient was discharged on day 35 postoperation at New York Heart Association (NYHA) class I after removing oxygen. It has now been 2 years since the operation, and she is still doing well.

## Discussion and conclusion

Early surgical correction has been established as the best treatment for TOF. Without surgical correction, most patients die during childhood [1], making adult patients with uncorrected TOF rare. However, there was a report of uncorrected TOF in the oldest patient aged 86 years [2].

There have been reports of adults with tetralogy of Fallot developing infective endocarditis and brain abscess, but surgical intervention has been avoided [3, 4]. There have only been two reports of repair surgery for tetralogy of Fallot in elderly patients aged 75 years or older, but in both cases the chief complaints were cyanosis and dyspnea [5, 6]. This case of a 78-year-old patient was an extremely rare case in which surgical intervention was indicated due to cyanosis and infective endocarditis.

Most patients with TOF have cyanosis from birth or the first year of life because of right-to-left shunting. If resistance to flow through the RVOT is less than resistance to flow through the aorta, there will be left-to-right shunt flow across the VSD without peripheral cyanosis and there will be a late presentation similarly to our case. Additionally, the patient has suffered from hypertension since her 40s and had high peripheral vascular resistance, which was one possible reason for the delayed cyanosis appearance. Furthermore, hemodynamic evaluation was performed only with four-dimensional (4D) flow MRI, and blood flow evaluation via collateral blood circulation was not considered. Therefore, there may be a discrepancy with the evaluation by cardiac catheterization. Considering the preoperative clinical findings and postoperative left heart failure, we believe that there may have been a large amount of blood flow from small collateral circulation channels.

There have been several reports on the surgical outcome of intracardiac repair for adult TOF. Sadiq *et al*. showed acceptable morbidity and mortality rates with good long-term surgical outcome [7], whereas Hörer *et al*. showed a high early mortality rate (15.4%) [8]. Opinions were also divided on the use of transannular patching (TAP), with Sadiq *et al*. concluding that TAP is not a risk factor, whereas Hörer *et al*. concluded that TAP is a risk factor [7, 8]. Considering the patient’s life expectancy and the preoperative pulmonary valve annulus system being 20 mm, we decided to form an outflow tract of up to 20 mm without TAP.

Despite the sufficient preoperative left ventricular volume and the restriction of RVOT formation, the increase in the left ventricular volume and pleural effusion after surgery required control of left heart failure. Although premised on improving hemodynamics, it appeared that the strategy for outflow tract formation similar to that in childhood should be reconsidered for the elderly. In our case, we performed complete surgical correction of TOF at 78 years of age, and the development of infective endocarditis was the definitive indication for surgery at rare old age.

### Supplementary Information

**Additional file 1: Video S1:** Magnetic resonance imaging scan of the heart revealing right ventricular outflow tract stenosis (wall shear stress: RA + RV + PA). **Video S2:** Magnetic resonance imaging scan of the heart revealing right ventricular outflow tract stenosis (energy loss: RA + RV + PA).

## Data Availability

Not applicable.

## Abbreviations

VSD: Ventricular septal defect
RVOT: Right ventricular outflow tract
TOF: Tetralogy of Fallot
