# Supplementary figures and images for: Surgical repair of tetralogy of Fallot in a 78-year-old woman: a case report
Source: J Med Case Rep. 2024 Mar 4;18:128. doi: 10.1186/s13256-024-04414-5 (PMC10910697; doi:10.1186/s13256-024-04414-5)

## Slide 1
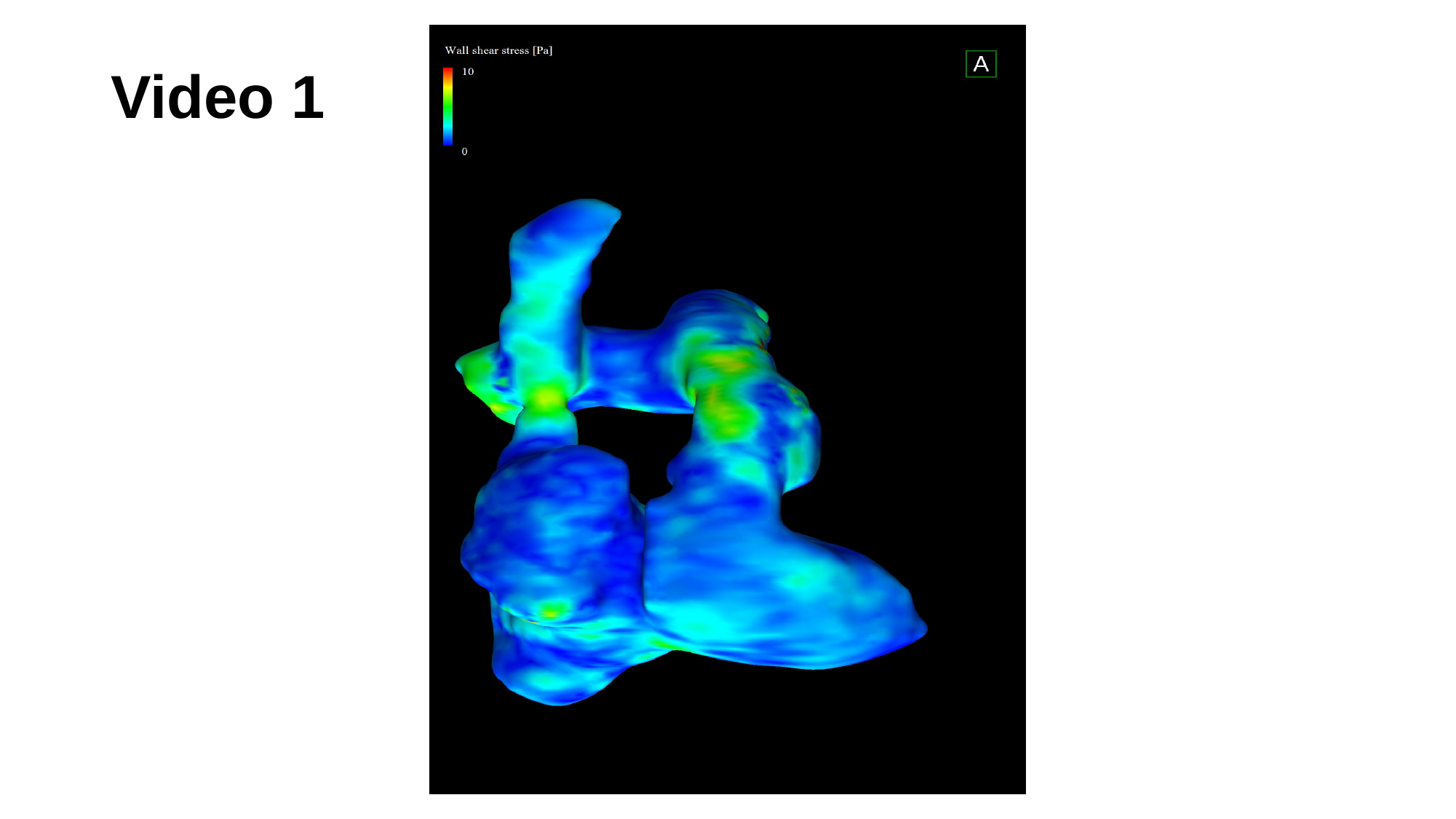

# Video 1

## Slide 2
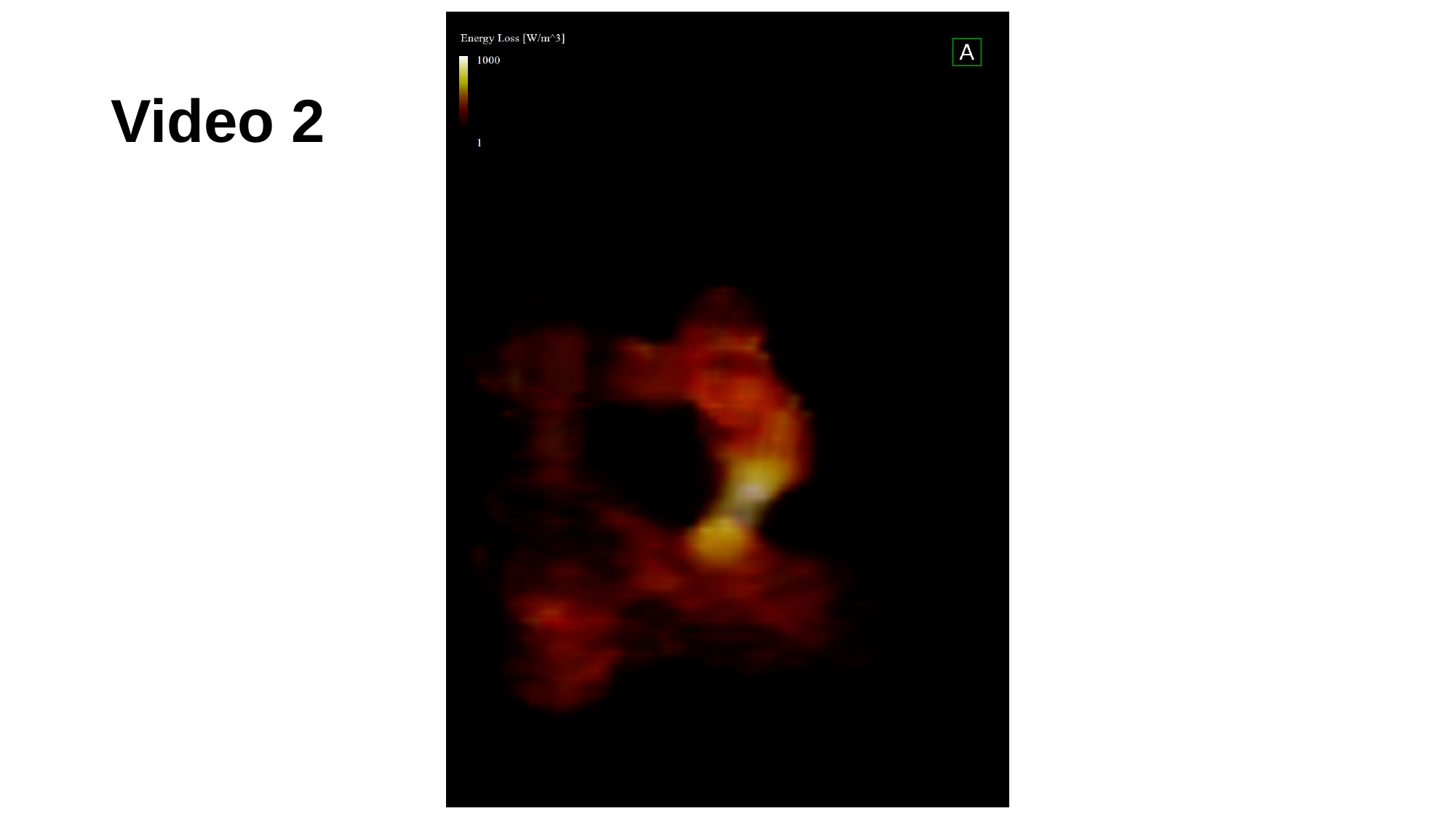

# Video 2

Supplement: Supplementary file 1 — Additional file 1: Video S1: Magnetic resonance imaging scan of the heart revealing right ventricular outflow tract stenosis (wall shear stress: RA + RV + PA). Video S2: Magnetic resonance imaging scan of the heart revealing right ventricular outflow tract stenosis (energy loss: RA + RV + PA). [file 13256_2024_4414_MOESM1_ESM.pptx]
